# Is total thyroidectomy with bilateral central neck dissection the only surgery for papillary thyroid carcinoma patients with clinically involved central nodes?

**DOI:** 10.1186/s12893-022-01699-5

**Published:** 2022-06-29

**Authors:** Kyorim Back, Jiyeon Lee, Anna Cho, Jun-Ho Choe, Jung-Han Kim, Young Lyun Oh, Jee Soo Kim

**Affiliations:** 1 Division of Endocrine Surgery, Department of Surgery, Chung-Ang University Hospital, Chung-Ang University School of Medicine, Seoul, South Korea; 2 Division of Endocrine Surgery, Department of Surgery, Samsung Medical Center, School of Medicine, Sungkyunkwan University, 81 Irwon-ro, Gangnam-gu, Seoul, 06351 South Korea; 3 Department of Pathology and Translational Genomics, Samsung Medical Center, School of Medicine, Sungkyunkwan University, Seoul, South Korea

**Keywords:** Papillary thyroid carcinoma, cN1a, Surgical strategy for cN1a PTC patients

## Abstract

**Background:**

In clinical practice, we often observed that patients who underwent total thyroidectomy due to clinically involved nodal disease (cN1a) actually had less extensive CLNM on final pathology. This study investigates whether total thyroidectomy and therapeutic bilateral CND are necessary for all PTC patients with cN1a.

**Methods:**

This study retrospectively reviewed 899 PTC patients who underwent total thyroidectomy with bilateral CND from January 2012 to June 2017. The patients were divided into two groups according to pre-operative central lymph node (CLN) status: cN0, no suspicious CLNM; cN1a, suspicious CLNM. We compared the clinicopathological features of these two groups.

**Results:**

There was no significant difference in recurrence between cN0 and cN1a groups after a mean follow-up time of 59.1 months. Unilateral cN1a was related to the largest central LN size ≥ 2 mm (OR = 3.67, p < 0.001) and number of CLNM > 5(OR = 2.24, p = 0.006). On the other hand, unilateral cN1a was not associated with an increased risk of contralateral lobe involvement (OR = 1.35, p = 0.364) and contralateral CLNM (OR = 1.31, p = 0.359). Among 106 unilateral cN1a patients, 33 (31.1%) were found to be pN0 or had ≤ 5 metastatic CLNs with the largest node smaller than 2 mm.

**Conclusions:**

Most cN1a patients were in an intermediate risk group for recurrence and required total thyroidectomy. However, lobectomy with CND should have performed in approximately 30% of the cN1a patients. Pre-operative clinical examination, meticulous radiologic evaluation, and intra-operative frozen sections to check the nodal status are prerequisites for this approach.

## Background

According to the 2015 American Thyroid Association (ATA) guidelines, total thyroidectomy with therapeutic central-compartment neck dissection (CND) is recommended for papillary thyroid carcinoma (PTC) patients with suspicious central lymph node metastasis (CLNM) on preoperative imaging [1]. Total thyroidectomy can be also considered when more than five metastatic central lymph node are present or the maximal dimension of a metastatic lymph node is greater than 2 mm, as these pathologic features indicate an intermediate risk of disease recurrence [2].

In spite of advantages after total thyroidectomy the availability of radioactive iodine (RAI) for ablation therapy and thyroglobulin as a follow-up tumor marker, several current studies have reported little or no difference in long-term rates of recurrence between lobectomy and total thyroidectomy [3, 4]. In addition, total thyroidectomy is associated with increased surgery-related complications [5, 6] and a requirement for lifelong thyroid hormone replacement therapy.

We hypothesized that, to a certain point, clinically involved central lymph node metastasis (cN1a) may not represent extensive metastasis, and lobectomy with ipsilateral CND can therefore be performed in selected cN1a PTC patients.

## Methods

### Patient selection

**Fig. 1:**
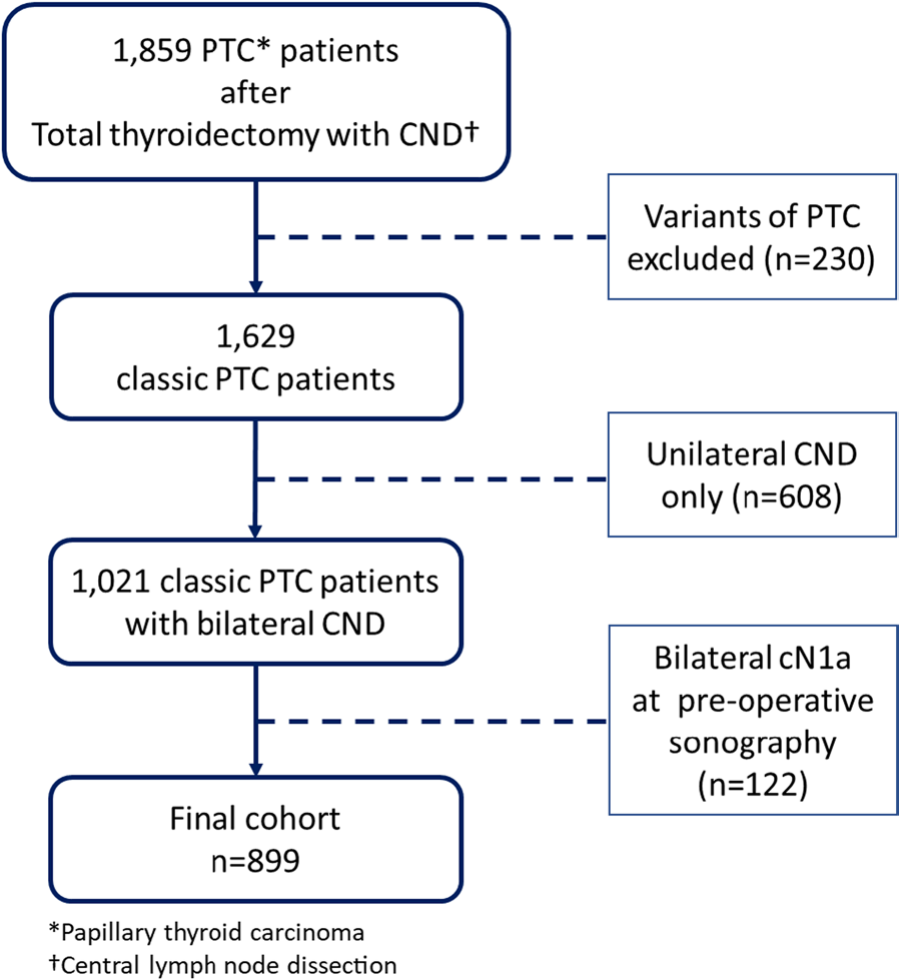
Flow chart of patient selection

This retrospective single-center study was approved by the Institutional Review Board at Samsung Medical Center. From January 2012 to June 2017, a total of 1859 patients diagnosed with PTC underwent total thyroidectomy with CND at the Thyroid Cancer Center of the Samsung Medical Center, which is a tertiary referral center in Korea. Among these, 230 cases with other variants of PTC (follicular variant, tall cell, cribriform-morular, etc.) were excluded, and 608 out of 1629 patients who had only unilateral CND were excluded, either. After excluding 122 patients who were suspicious of bilateral cN1a at pre-operative sonographic evaluation, a total of 899 patients were included in the final cohort (Fig. 1).

All patients were assessed preoperatively by ultrasonography (US) to evaluate primary tumors and identify abnormal lymph nodes. Suspicious thyroid lesions were diagnosed by fine-needle aspiration (FNA). Clinically involved lymph node disease (cN1a) was defined as lymph node metastases in the central neck compartment on preoperative US.

### Surgical methods

Surgical strategies were performed according to the ATA guidelines [1]. Total thyroidectomy and therapeutic CND is typically performed after CLNM is detected during preoperative US. Patients who were node-negative at the time of preoperative ultrasonography underwent total thyroidectomy with prophylactic CND at the surgeon’s discretion. CND was defined as a central neck dissection extending superiorly to the hyoid bone, inferiorly to the innominate artery, laterally to the carotid sheaths, and dorsally to the prevertebral fascia. The term “ipsilateral” was used to indicate the same side as the main tumor, and “contralateral” was defined as the opposite side of the main tumor. In cases of bilateral tumors, the largest tumor was considered to be the main tumor. Central-compartment lymph nodes include pretracheal, prelaryngeal, and paraesophageal lymph nodes. For example, ipsilateral CLNs indicate pretracheal, prelaryngeal, and paraesophageal lymph nodes in the same side as the main tumor.

### Histopathological examination of surgical specimens

Surgical specimens were examined microscopically by at least two experienced pathologists. The following histopathologic factors were assessed: main tumor size (longest diameter of the largest lesion), main tumor cell type, multifocality, bilaterality, microscopic or gross extrathyroidal extension (ETE), regional lymph node metastasis (central or lateral compartment), and underlying conditions of the thyroid, such as chronic lymphocytic thyroiditis (CLT). We checked the maximum dimension of metastatic foci in the lymph nodes if metastasis was present. With multiple tumor deposits in a single lymph node, the pathologists measured the largest dimension of the largest deposit. Multifocality was defined as the presence of two or more lesions in one lobe, regardless of bilaterality. ETE was defined as direct extension to perithyroidal soft tissues, strap muscles, nerves, or small vascular structures by the thyroid tumor. Minimal (microscopic) ETE was defined as an invasion detected only in a microscopic exam. If the involved tissue was completely removed along with the tumor, the resection margin was reported as negative. Staging of thyroid cancer was determined in accordance with the 7th edition of the American Joint Committee on Cancer’s Cancer Staging Manual and the Future of TNM [7].

### Post-operative follow-up and management

After the initial surgery, all patients underwent regular follow-up at 6- to 12-month intervals with clinical evaluations including physical examinations, US, thyroid function tests (including T3, free T4, thyroid-stimulating hormone), and measurements of serum thyroglobulin (Tg) with its antibody. CT or iodine-131 (^131^I) scans was administrated if needed during follow up. Suspicious lesions for recurrence were evaluated by US-guided FNA biopsy with or without washout-thyroglobulin(wTg) levels and/or CT or positron emission tomography. Locoregional recurrence was defined as the presence of tumors or metastatic lymph nodes on cytology from FNA or the elevation of wTg level. RAI therapy was performed with ^131^I at 4–12 weeks after surgery according to ATA guidelines.^1^ RAI was administered after thyroid hormone withdrawal or after stimulation with recombinant thyroid-stimulating hormone. When RAI treatment was no longer required, patients resumed regular follow-ups.

### Statistical analysis

Statistical analysis was performed using IBM SPSS version 24.0. Variables with a *p* value < 0.05 were considered statistically significant. The chi-square test and Student’s t-test were used to compare categorical variables between the group of unilateral cN1a patients and the group of cN0 patients. For factors that appeared to be related based on initial analysis, we used logistic regression to identify associated variables.

## Results

### Clinicopathological characteristics of 899 PTC patients

The 899 patients were divided into two groups according to preoperative physical and radiologic CLN status: 793 (88.2%) patients without clinically involved CLN (cN0) and 106 (11.8%) patients with clinically involved CLN (cN1a). The clinicopathological characteristics of the patients in each group are listed in Table 1. The mean age was significantly lower in the cN1a group (cN0: cN1a = 47.1 years: 43.4 yrs.; *p* = 0.002). Association with chronic lymphocytic thyroiditis was more common in the cN1a group (*n* = 44; 41.5%) than in the cN0 group (*n* = 230; 29%) (*p* = 0.009). The mean number of retrieved CLNs was 9.7 in the cN0 group and 11.7 in the cN1a group (*p* < 0.001). CLNM was more frequent in the cN1a group compared to the cN0 group (77.4% vs. 57.5%; *p* < 0.001). In contrast, contralateral central neck metastasis was not significantly different; 34.9% in the cN0 group and 37.8% in the cN1a group (*p* = 0.609). We analyzed the number and largest diameter of metastatic LNs in patients with pathologically proven CLNM (pN1, *n* = 538). The number of patients with more than 5 metastatic LNs was 81 (17.8%) in the cN0 group and 26 (31.7%) in the cN1a group, and this result was statistically significant (*p* = 0.004). There were more cases with largest LN diameter ≥ 2 mm in the cN1a group (82.8%) than in the cN0 group (66.7%) (*p* < 0.001). With a median follow-up of 59.1 months (range, 24 to 98 months), there was no difference in recurrence between the two groups (p = 1.000); 14 patients (1.8%) in the cN0 group and 2 patients (1.9%) in the cN1a group (Table 1).

**Table 1:** Pathological tumor characteristics of cN0 and unilateral cN1a PTC patients

Pathological characteristics	cN0 no. (%)	Unilateral cN1a no. (%)	*p*-value
Total (n = 899)	793	106	
Sex			
Female	605 (76.3)	81 (77.4)	0.808
Male	188 (23.7)	25 (22.6)	
Age (year)			
Mean ± SD	47.1 ± 11.9	43.4 ± 10.7	0.002
< 55	565 (71.2)	91 (85.8)	
≥ 55	222 (28.8)	15 (14.2)	
Main tumor laterality			
Right	448 (56.5)	43 (40.6)	0.002
Left	345 (43.5)	63 (59.4)	
Tumor size (cm)			
Mean ± SD	1.17 ± 0.7	1.20 ± 0.5	0.603
≤ 1.0	392 (49.4)	44 (41.5)	
1.0–2.0	326 (41.1)	56 (52.8)	
2.0–4.0	70 (8.8)	6 (5.7)	
> 4.0	5 (0.6)	0 (0)	0.611
Bilaterality			
Absent	548 (69.1)	71 (67.0)	0.657
Present	245 (30.9)	35 (33.0)	
Multifocality			
Absent	592 (74.7)	70 (66.0)	0.059
Present	201 (25.3)	36 (34.0)	
BRAF mutation (n = 634)			
Absent	75 (13.1)	9 (14.8)	0.715
Present	498 (86.9)	52 (85.2)	
Extrathyroidal extension			
Absent	277 (34.9)	42 (39.6)	0.343
Present	516 (65.1)	64 (60.4)	
Chronic lymphocytic thyroiditis			
Absent	563 (71.0)	62 (58.5)	0.009
Present	230 (29.0)	44 (41.5)	
Number of retrieved central LNs (no.)			
Mean (range) ± SD	9.7 (1.0–34.0) ± 5	11.7 (2.0–36.0) ± 6	< 0.001
Central LN metastasis			
Absent	337 (42.5)	24 (22.5)	< 0.001
Present	456 (57.5)	82 (77.4)	
Ipsilateral central neck metastasis			
Absent	36 (7.9)	2 (2.4)	0.076
Present	420 (92.1)	80 (97.6)	
Contralateral central neck metastasis*			
Absent	297 (65.1)	51 (62.2)	0.609
Present	159 (34.9)	31 (37.8)	
Number of metastatic CLNs (no.)			
Mean(range) ± SD	2.22(1.0–20.0) ± 2.8	2.24(1.0–15.0) ± 2.9	0.004
≦ 5	375 (82.2)	56 (68.3)	
> 5	81 (17.8)	26 (31.7)	
Retrieved CLNM size (mm)			
< 2.0	152 (33.3)	10 (12.2)	< 0.001
≧ 2.0	304 (66.7)	72 (82.8)	
Loco-regional recurrence			
Absent	779 (98.2)	104 (98.1)	1.000
Present	14 (1.8)	2 (1.9)	

*PTC* papillary thyroid carcinoma, *cN0* no clinically involved central lymph node, *cN1a* clinically involved central lymph node, *no* number, *SD* standard deviation, *LN* lymph node, *CLNM* central lymph node metastasis

*Patients with ipsilateral central neck metastasis and contralateral central neck metastasis simultaneously OR with contralateral central neck metastasis only were counted

### Logistic analysis for factors related to contralateral lobe involvement & contralateral central lymph node metastasis

We performed a multivariate analysis with 643 patients who were pre-operatively diagnosed with unilateral PTC to verify factors related to contralateral lobe involvement. Multifocality was significantly associated with contralateral lobe involvement (OR = 3.164, *p* < 0.001), while unilateral cN1a was not (*p* = 0.364) (Table 2).

**Table 2:** Associations between contralateral lobe involvement^a^ and clinicopathological characteristics of PTC patients (*n* = 643^b^)

Clinicopathological characteristics	Univariate analysis			Multivariate analysis		
	Adjusted OR	95% CI	*p*-value	Adjusted OR	95% CI	*p*-value
Unilateral cN1a (Ref = cN0)	1.348	0.708–2.567	0.364			
Male sex (Ref = female)	0.813	0.472–1.401	0.456			
Patient age (per 10 years)	1.018	0.999–1.036	0.063	1.015	0.996–1.034	0.134
Tumor size (per 0.1 cm)	0.961	0.678–1.362	0.823			
Multifocality (Ref = absent)	3.262	2.006–5.305	< 0.001	3.164	1.942–5.157	< 0.001
ETE (Ref = absent)	0.772	0.486–1.226	0.273			
Chronic lymphocytic thyroiditis (Ref = absent)	1.320	0.813–2.141	0.261			
Ipsilateral central neck metastasis (Ref = absent)	0.707	0.447–1.116	0.136			

*PTC* papillary thyroid carcinoma, *cN0* no clinically involved central lymph node, *cN1a* clinically involved central lymph node, *ETE* extrathyroidal extension, *OR* odds ratio, *CI* confidence interval, *Ref* reference

^a^Incidentally found contralateral lobe PTMC on permanent pathology

^b^Patients with preoperatively detected bilateral PTC were excluded

Further subgroup analysis including patients with pathologically-proven unilateral PTC (*n* = 619) indicated that factors of male sex (OR = 1.798, *p* < 0.010), tumor size (OR = 1.605, *p* = 0.001), multifocality (OR = 2.496, *p* < 0.001), and ipsilateral central neck metastasis (OR = 3.255, *p* < 0.001) increased the risk of contralateral central neck metastasis. Unilateral cN1a, however, was not significantly associated with contralateral central neck metastasis (*p* = 0.359) (Table 3).

**Table 3:** Associations between contralateral central neck metastasis and clinicopathological characteristics of PTC patients (*n* = 619^a^)

Clinicopathological characteristics	Univariate analysis			Multivariate analysis		
	Adjusted OR	95% CI	*p*-value	Adjusted OR	95% CI	*p*-value
Unilateral cN1a (Ref = cN0)	1.672	0.970–2.882	0.064	1.312	0.734–2.342	0.359
Male sex (Ref = female)	2.254	1.485–3.421	< 0.001	1.798	1.153–2.801	0.010
Patient age (per 10 years)	0.965	0.948–0.982	< 0.001	0.975	0.957–0.994	0.009
Tumor size (per 0.1 cm)	1.661	1.270–2.173	< 0.001	1.605	1.200–2.145	0.001
Multifocality (Ref = absent)	2.056	1.314–3.215	0.002	2.496	1.539–4.046	< 0.001
ETE (Ref = absent)	0.998	0.664–1.501	0.992			
Chronic lymphocytic thyroiditis (Ref = absent)	0.808	0.516–1.266	0.352			
Ipsilateral central neck metastasis (Ref = absent)	4.149	2.493–6.903	< 0.001	3.255	1.901–5.574	< 0.001

*PTC* papillary thyroid carcinoma, *cN0* no clinically involved central lymph node, *cN1a* clinically involved central lymph node, *ETE* extrathyroidal extension, *OR* odds ratio, *CI* confidence interval, *Ref* reference. ^a^Patients with only unilateral PTC were included, and patients with incidentally found contralateral lobe PTMC on permanent pathology were excluded

### Risk factors for the number of metastatic central lymph node > 5 & the largest metastatic central lymph node ≥ 2 mm

Table 4 shows that the risk factors for more than five metastatic LNs in pN1 patients were unilateral cN1a (OR = 2.240, *p* = 0.006), tumor size (OR = 1.613, *p* = 0.003), multifocality (OR = 1.799, *p* = 0.022), and contralateral central neck metastasis (OR = 3.550, *p* < 0.001). Table 5 indicated that the risk factors for a metastatic LN larger than 2 mm were unilateral cN1a (OR = 3.667, *p* < 0.001) and age (OR = 0.946, *p* < 0.001).

**Table 4:** Associations between number of CLNM > 5 and clinicopathological characteristics of PTC patients with pathologically-proven pN1a (*n* = 538)

Clinicopathological characteristics	Univariate analysis			Multivariate analysis		
	Adjusted OR	95% CI	*p*-value	Adjusted OR	95% CI	*p*-value
Unilateral cN1a (Ref = cN0)	2.149	1.273–3.628	0.004	2.240	1.260–3.979	0.006
Male sex (Ref = female)	1.538	0.989–2.393	0.056			
Patient age (per 10 years)	0.966	0.948–0.985	0.001	0.968	0.948–0.988	0.002
Tumor size (per 0.1 cm)	1.742	1.313–2.311	< 0.001	1.613	1.180–2.974	0.003
Multifocality (Ref = absent)	2.030	1.296–3.180	0.002	1.799	1.088–2.974	0.022
ETE (Ref = absent)	1.631	1.010–2.632	0.045	1.607	0.956–2.699	0.073
Chronic lymphocytic thyroiditis (Ref = absent)	1.083	0.675–1.739	0.740			
Contralateral lobe involvement (Ref = absent)	1.092	0.689–1.729	0.709			
Contralateral central neck metastasis (Ref = absent)	4.416	2.827–6.898	< 0.001	3.550	2.215–5.690	< 0.001

*PTC* papillary thyroid carcinoma, *CLNM* central lymph node metastasis, *cN0* no clinically involved central lymph node, *cN1a* clinically involved central lymph node, *ETE* extrathyroidal extension, *OR* odds ratio, *CI* confidence interval, *Ref* reference

**Table 5:** Associations between maximum CLNM size ≥ 2 mm and clinicopathological characteristics of PTC patients with pathologically-proven pN1a (n = 538)

Clinicopathological characteristics	Univariate analysis			Multivariate analysis		
	Adjusted OR	95% CI	*p*-value	Adjusted OR	95% CI	*p*-value
Unilateral cN1a (Ref = cN0)	3.600	1.807–7.174	< 0.001	3.667	1.803–7.460	< 0.001
Male sex (Ref = female)	0.987	0.662–1.471	0.949			
Patient age (per 10 years)	0.946	0.931–0.962	< 0.001	0.946	0.930–0.962	< 0.001
Tumor size (per 0.1 cm)	1.174	0.886–1.555	0.265			
Multifocality (Ref = absent)	1.035	0.681–1.574	0.872			
ETE (Ref = absent)	1.117	0.758–1.646	0.577			
Chronic lymphocytic thyroiditis (Ref = absent)	1.166	0.764–1.779	0.476			
Contralateral lobe involvement (Ref = absent)	1.117	0.743–1.681	0.595			
Contralateral central neck metastasis (Ref = absent)	1.329	0.897–1.970	0.157			

*PTC* papillary thyroid carcinoma, *CLNM* central lymph node metastasis, *cN0* no clinically involved central lymph node, *cN1a* clinically involved central lymph node, *ETE* extrathyroidal extension, *OR* odds ratio, *CI* confidence interval, *Ref* reference

### Final pathology of patients with unilateral cN1a

In 106 unilateral cN1a patients, 33 (31.1%) were found to be pN0 or had ≤ 5 metastatic CLNs with the largest node smaller than 2 mm. For the balance of the patients (69.2%), 1 patient (0.9%) had more than 5 metastatic CLNs with the largest node < 2 mm or pN0, 47 patients (44.3%) had fewer than 5 metastatic CLNs with the largest node ≥ 2 mm, and 25 patients (23.7%) had more than 5 metastatic CLNs with the largest node ≥ 2 mm (Table 6).

**Table 6:** Central lymph node status on final pathology of unilateral cN1a PTC patients

Node status	*N* = 106 *n* (%)
≤ 5 pN1micrometastasis + < 0.2 cm in largest dimension or pN0	33 (31.1%)
> 5 pN1micrometastasis + < 0.2 cm in largest dimension	1 (0.9%)
≤ 5 pN1micrometastasis + ≥ 0.2 cm in largest dimension	47 (44.3%)
> 5 pN1micrometastasis + ≥ 0.2 cm in largest dimension	25 (23.7%)

*PTC* papillary thyroid carcinoma, *n* number, *pN0* no central lymph node metastasis in final pathology, *pN1a* pathologically-proven central lymph node metastasis, *cN1a* clinically involved central lymph node

## Discussion

For many decades, cN1a patients were expected to have more than five metastatic central LNs and/or the largest metastatic LN size greater than 2 mm, and total thyroidectomy and CND are considered because of the recurrence rate up to 20% [1, 8]. However, among cN1a patients who had total thyroidectomy and CND, some had less extensive or even no central neck metastasis on final pathology. This study was designed based on the results from previous study by our research team [9]. Among 295 papillary thyroid microcarcinoma patients with cN1a, about 73% of patients actually had micrometastasis and these low-risk patients shouldn’t have undergone total thyroidectomy and bilateral CND [9]. Since surgery has been decreasing in patient with tumor size less than 1 cm due to active surveillance, we wondered what results would be in patients with all sizes of PTC. We considered that including all tumor size will be more useful in real world practice. To improve a quality of study, we collected more sample size from different period and performed more test statistics this time. This study searched for the patients with cN1a disease who are candidates for lobectomy with unilateral CND rather than total thyroidectomy with bilateral CND by comparing patients with cN0 and cN1a.

The accuracy of preoperative diagnosis of LN status has improved markedly with the advent of high-resolution sonography and increased sonographer experience [10–12]. While identification of cN1a patients has improved with these advancements, detection of microscopic CLNM remains challenging [13]. In clinical practice, we often observed that patients who underwent total thyroidectomy due to cN1a actually had less extensive CLNM on final pathology. In these patients, a less-extensive surgery rather than total thyroidectomy may have been sufficient. We therefore hypothesized that lobectomy with unilateral CND instead of total thyroidectomy with bilateral CND could be safely performed for the selected patients with cN1a.

To support this hypothesis, several conditions should be considered. First, cN1a status does not always indicate having more than five metastatic LNs or largest metastatic LN size greater than 2 mm. Second, patients with a cN1a are not at increased risk of contralateral lobe involvement. Third, a cN1a patient does not have increased contralateral CLNM. Finally, intra-operative frozen exam should be available during the operation.

As described above, this study showed that cN1a was closely associated with more than five metastatic LNs or largest metastatic LN size greater than 2 mm, and approximately 69.0% of the patients required total thyroidectomy. However, the other 31.1% of patients (*n* = 33) had fewer than 5 metastatic CLNs with the largest metastatic LN size less than 2 mm, indicating that total thyroidectomy could have avoided. Furthermore, 13 of 33 patients did not have any lymph node metastasis. It is possible that lymph node enlargement with suspicious feature could be caused by a local benign condition [14] (e.g., underlying parenchymal disease). In patients with CLT, ultrasound features can be diverse depending on the severity and the phase of disease [15, 16]. CLT with CLN enlargement is common in Korean patients who live in iodine-rich areas [17, 18]. Among above 33 cN1a patients who had fewer than 5 metastatic CLNs with the largest node < 2 mm, 22 patients (67.0%) presented with CLT in the final pathology. This condition is the most common inflammatory disorder of the thyroid gland [19], and although its influence on PTC prognosis remains controversial [20], many authors maintain that coexistence of CLT with PTC is related to lower-stage disease and favorable prognostic factors [21–24]. If patients with cN1a have underlying thyroiditis, an intra-operative frozen exam, if available, is helpful to avoid unnecessary contralateral lobectomy and contralateral CND.

Despite a controversy over usefulness of frozen exam, several studies showed that intra-operative frozen exam had the effectiveness and clinical value [25, 26]. In addition, gross inspection by surgeons is not a precise method for differentiating CLNM [27]. Although this usually takes additional time and has limited accuracy of diagnosis, it is obvious that intraoperative frozen exam is more precise than intraoperative gross inspection or palpation. Intra-operative frozen biopsy can be helpful for reducing completion thyroidectomy or unnecessary overtreatment. However, comprehensive evaluation of whole dissected CLN is not feasible and recommendable in every practice. Collaborative discussions with pathologists can address these issues.

Although there is no consensus about the extent of CND in unilateral cN1a patients, many studies suggest that prophylactic contralateral CND is unnecessary [28–31] because of the low probability of contralateral CLNM and potential morbidity that can accompany an extensive surgery [32, 33]. Prophylactic contralateral CND was associated with increased rates of transient and permanent hypoparathyroidism [34, 35]. Our results indicate that unilateral cN1a does not increase the risk of contralateral lobe involvement or contralateral central neck metastasis, which means that contralateral CND may not be needed for unilateral cN1a patients. The clinical significance of contralateral CND remains unclear especially when contralateral CLNM is a microscopic. Not shown in this study, recurrence rate of patients who underwent total thyroidectomy with unilateral CND was only 2.6%, comparable to 1.9% in the recurrence rate of patients who underwent total thyroidectomy with bilateral CND. Further studies are required to confirm the necessity of prophylactic contralateral CND, as well as the relationships between contralateral CLNM and number, maximum size, and ipsilateral CLNM involvement.

There were several limitations of this study, which was a non-randomized, retrospective cohort study at a single institution. First, potential confounding variables may not have been identified. Second, subgroup analysis comparing pathologic nodal status and clinical nodal status was not performed. Since this study was retrospective study, difference between clinical nodal status and pathologic nodal status was one of the unavoidable discrepancies caused by radiologist’s or pathologist’s experience. Third, inter-observer variation in the detection and interpretation of cervical lymph node metastasis and inconsistent surgical management were involved because of the long-term follow-up. Lastly, our findings may not be applicable to all centers because availability and cooperation of skilled pathologists are essential for surgical decision-making. In spite of these limitations, our retrospective data showed that about 31% of patients with unilateral cN1a were able to avoid total thyroidectomy. Prospective study with larger cohort is essential to clarify this subject.

## Conclusion

This study suggests that, although the majority of PTC patients with unilateral cN1a still needs total thyroidectomy with bilateral CND, lobectomy and unilateral CND can be considered in selected patients. Intra-operative frozen exam may help to identify low risk patients among cN1a patients and reduce overtreatment.

## Data Availability

The data that support the findings of this study are available from Samsung Medical Center but restrictions apply to the availability of these data, which were used under license for the current study, and so are not publicly available. Data are however available from the authors upon reasonable request and with permission of Samsung Medical Center.

## Abbreviations

CND: Central central-compartment neck dissection
PTC: Papillary thyroid carcinoma
CLNM: Central lymph node metastasis
cN1a: Clinically involved central lymph node disease
cN0: Clinically not involved central lymph node disease
CLN: Central lymph node
RAI: Radioactive iodine
Tg: Serum thyroglobulin
wTg: Washout-thyroglobulin
US: Ultrasonography
FNA: Fine-needle aspiration
ETE: Extrathyroidal extension
CLT: Chronic lymphocytic thyroiditis
